# EEG activity over ipsilateral and contralateral M1 during simple and complex hand tasks: variations with motor learning

**DOI:** 10.3389/fnins.2025.1681250

**Published:** 2025-11-06

**Authors:** Jun Zhao, Yifan Wang, Dengzhe Hou, János Négyesi, De-Lai Qiu, Ryoichi Nagatomi

**Affiliations:** 1 Brain Science Institute, Jilin Medical University, Jilin Street, Jilin City, Jilin, China; 2 Department of Medicine and Science in Sports and Exercise, Tohoku University Graduate School of Medicine, Seiryo-machi, Aoba-ku, Sendai, Miyagi, Japan; 3 Research Institute of Electrical Communication, Tohoku University, Katahira, Aoba Ward, Sendai, Japan; 4 Department of Kinesiology, Hungarian University of Sports Science, Budapest, Hungary; 5 Neurocognitive Research Center, National Institute of Mental Health, Neurology and Neurosurgery, Budapest, Hungary; 6 CRU Hungary Ltd., Budapest, Hungary; 7 Designing Future Health Initiative (DFHI), Promotion Office of Strategic Innovation, Aramaki Aza Aoba, Aoba-ku, Sendai, Japan; 8 Division of Biomedical Engineering for Health & Welfare, Tohoku University Graduate School of Biomedical Engineering, Aramaki Aza Aoba Aoba-ku, Sendai, Miyagi, Japan

**Keywords:** ipsilateral M1 activation, motor learning, complexity, handedness, EEG

## Abstract

**Introduction:**

The functional role of the ipsilateral primary motor cortex (iM1) activation in motor skill acquisition is widely researched; however, its interaction with task complexity remains unclear. This study aimed to address a critical gap in motor neuroscience: how the electroencephalogram (EEG) activation dynamics (specifically in the gamma frequency band) recorded by electrodes over the contralateral primary motor cortex (cM1) and iM1 evolve during the acquisition of simple vs. complex motor skills, and whether these dynamics are modulated by hand dominance.

**Methods:**

In a randomized controlled trial, 48 right-handed participants were randomly assigned to train on either simple or complex visuomotor tasks using their right (SR, CR, respectively) or left hand (SL, CL, respectively), with 12 participants per group. One participant in the SL group was excluded due to poor EEG quality, resulting in 11 participants in the SL group. Participants completed 10 training blocks followed by skill retention tests. Brain activity was recorded continuously via 64-channel EEG.

**Results:**

Data from 47 participants revealed that, prior to training, the high-gamma band (50-80Hz) activation recorded by electrodes over iM1 exhibited significantly higher activation during simple tasks compared to complex tasks, irrespective of the hand used. However, after 10 training sessions, the electrodes over iM1 activation increased during complex tasks but decreased during simple task for both hands, eliminating significant differences in activation levels between simple and complex tasks. Furthermore, no significant changes were observed in the EEG activation recorded by electrodes over cM1 before and after training.

**Conclusions:**

Our data indicated that task complexity affects the EEG activation identified from electrodes over iM1. Specifically, complex task training for both right and left hands enhanced the high-gamma frequency band power recorded from the electrodes over iM1. These findings highlight differential neural responses within specific frequency bands, potentially reflecting distinct impacts of the interventions applied to each group. This supports the idea that iM1 plays a dynamic, task-dependent role in skill acquisition, consistent with prior proposals that iM1 activation scales with task demands.

## Introduction

The human motor system has long been anchored in the principle of contralateral control, wherein corticospinal fibers cross to regulate distal movements of the opposite body side. This framework, however, has been increasingly challenged by discoveries of primary motor cortex (M1) neurons active during both contralateral and ipsilateral hand movements, as revealed by single-cell recordings in primates (Tanji et al., 1988; Donchin et al., 2002). Such findings, consistently replicated across species and methodologies (Kim et al., 1993; Li et al., 1996; Kawashima et al., 1997; Singh et al., 1998; Cramer et al., 1999; Nirkko et al., 2001; Kobayashi et al., 2003), suggest a more complex picture of motor control than previously thought.

Post-stroke research has further complicated this landscape. Upregulation of contralateral M1 (cM1) activity may be crucial for improving paretic arm function post-stroke (Netz et al., 1997; Caramia et al., 2000). While post-stroke activity often increases abnormally in ipsilateral M1 (iM1), its functional role remains debated. On one hand, some studies interpret iM1 engagement as an adaptive compensation: the intact hemisphere recruits iM1 to offset damage in the lesioned hemisphere, thereby facilitating rehabilitation (Fries et al., 1993; Ward et al., 2003). On the other hand, some studies have shown that motor-evoked potentials are induced in ipsilateral muscles most commonly in patients with poor recovery (Turton et al., 1996), and some imaging studies have suggested that ipsilateral activation decreases as recovery occurs (Marshall et al., 2000; Carey et al., 2002; Feydy et al., 2002). These findings suggest that increased ipsilateral motor cortex involvement is a marker of poor outcome (Turton et al., 1996), rather than an adaptive response contributing to reduced impairment.

Notably, the controversy surrounding iM1 is not limited to pathological states but extends to normal motor learning. Early neuroimaging studies revealed asymmetric M1 activation during repetitive movements, with prominent ipsilateral engagement (Kim et al., 1993). Subsequent work elaborated that in right-handed individuals, left M1 activation is enhanced during non-dominant (left) hand use, with this effect amplifying as task complexity increases (Chen et al., 1997; Verstynen et al., 2005). TMS studies further linked iM1 excitability to skilled performance: Morishita et al. demonstrated heightened iM1 excitability during fine left-hand movements (e.g., chopstick use) (Morishita et al., 2011), while van den Berg et al. found that disrupting dominant M1 impairs complex ipsilateral finger tasks (van den Berg et al., 2011), suggesting iM1 activation supports performance. Conversely, other studies reported inhibitory effects, as Suzuki et al. observed suppressed iM1 excitability (reduced MEPs) alongside improved performance in a ball rotation task (Suzuki et al., 2013), and 1 Hz rTMS-induced iM1 inhibition prior to learning enhanced motor outcomes (Plewnia et al., 2003; Schambra et al., 2003; Kobayashi et al., 2004, 2009; Buetefisch et al., 2011). These conflicting results raise critical questions as to the significance and the role of M1 activation in motor learning.

Methodological limitations have hindered the resolution of these questions. fMRI studies (Haar et al., 2017) offer spatial precision but lack temporal granularity to capture real-time activation shifts during skill acquisition. TMS (Buetefisch et al., 2011) probes excitability but disrupts natural neural dynamics. Electrocorticography (ECoG) (Ganguly et al., 2009; Scherer et al., 2009) provides high spatiotemporal resolution but is invasive, restricting sample generalizability. This methodological gap underscores the need for non-invasive approaches with fine temporal resolution, such as electroencephalogram (EEG), to dissect the dynamic interplay of cM1 and iM1 during motor learning.

Although the role of the iM1 in motor control has been extensively studied using EEG, the dynamic changes and regulatory mechanisms of iM1 activation patterns during motor learning of simple vs. complex hand tasks still remain unclear. Previous studies either focused on static task comparisons or lacked analysis of frequency-specific changes during learning, failing to clarify the association between iM1 activation, task complexity, and learning stages. Alpha and theta bands are more closely related to motor learning than other frequency bands (van der Cruijsen et al., 2021). Additionally, theta oscillations are related to attentional demands (Fiebelkorn and Kastner, 2019) which are necessary components for skill acquisition. Importantly, gamma band oscillations have been established as a well-validated mediator of M1 function in this research domain. Evidence has shown that gamma oscillations in the rat M1 regulate motor learning (Otsuka and Kawaguchi, 2021). Moreover, high-gamma oscillations in the motor cortex demonstrate a strong correlation with movement rate, as trials with higher movement rates were associated with increased high-gamma power (Haverland et al., 2025). Growing evidence implicates gamma oscillations in motor function: high-gamma phase-amplitude coupling may induce long term potentiation-like plasticity in human M1 (Baur et al., 2022), gamma-frequency transcranial alternating current stimulation (gamma-tACS) might modulate transcallosal inhibition and balance interhemispheric interactions to promote motor recovery (Tingting et al., 2025), and deep brain stimulation (DBS)-induced gamma oscillations exert prokinetic effects in Parkinson's disease (Mathiopoulou et al., 2025). These findings collectively confirm gamma activity as a key index of M1 plasticity and interhemispheric communication, providing a robust methodological basis for investigating the role of iM1 in motor learning. Thus, our study aims to address a key gap in understanding: namely, how iM1 contributes to motor learning of simple vs. complex hand tasks, using gamma-band activity as the core indicator to quantify the involvement of iM1 in interhemispheric coordination. We propose a core hypothesis: Motor learning will enhance ipsilateral gamma-band activity specifically during complex tasks due to the continued demand for controlled processing, whereas simple tasks transition to automaticity with practice, diminishing the need for such integrative cortical involvement. The mechanistic basis for this hypothesis is that simple tasks easily form automatic processing through repetition (Schneider and Chein, 2003), requiring only unilateral motor control mediated by the cM1 and less interhemispheric coordination involving iM1 (Zhao et al., 2025). In contrast, complex tasks require sustained cognitive control and fine regulation of finger sequences, necessitating iM1 to synchronize neural activity via the gamma band to mediate interhemispheric information integration for skill acquisition (Baur et al., 2022; Tingting et al., 2025).

In our previous study analyzing the same dataset of inter limb transfer, we found complex tasks enhanced alpha and theta coherence in motor and sensorimotor areas (Zhao et al., 2025). The present study extends this work by specifically examining gamma band EEG activation recorded from the electrodes over cM1 and iM1 during simple and complex tasks, providing a complementary perspective on interhemispheric dynamics during motor skill acquisition.

## Methods

### Participants

As reported before (Zhao et al., 2025), a total of 48 healthy (23 males, 25 females), strong right-handed individuals, with a mean age of 25.81 years (*SD* = 2.70) and a mean handedness index of 98.04 (*SD* = 5.92), participated in this study. Handedness was assessed using the Edinburgh Handedness Inventory (Oldfield, 1971). Participants were randomly assigned to one of four groups: ‘SL: Simple task training for the left hand', ‘CL: Complex task training for the left hand', ‘SR: Simple task training for the right hand', and ‘CR: Complex task training for the right hand', with 12 participants in each group. One participant from the SL group was excluded due to poor EEG signal quality, resulting in a final count of 11 participants in that group, while the other groups each retained 12 participants. Demographic and handedness characteristics were comparable across groups (One-way ANOVA, age: F _(3, 46)_ = 0.137, *p* = 0.938, η^2^ = 0.009; handedness: F_(3, 46)_ = 0.388, *p* = 0.762, η^2^ = 0.026). All participants were free from neurological or orthopedic conditions, and no participants had prior piano-learning experience or were employed in computer-related professions. After receiving both verbal and written explanations of the study protocol, participants provided their informed consent as per the Declaration of Helsinki. The study protocols were approved by the Tohoku University Ethical Committee (Approval No. 2021-1-897).

### Experimental setup

As described in our previous work (Zhao et al., 2025), participants were comfortably seated approximately 1.1 meters from a computer screen in a dimly lit, electromagnetically shielded room. The experiments utilized a 19-inch IBM LCD display, and custom software was employed to present the stimuli.

**Figure 1:**
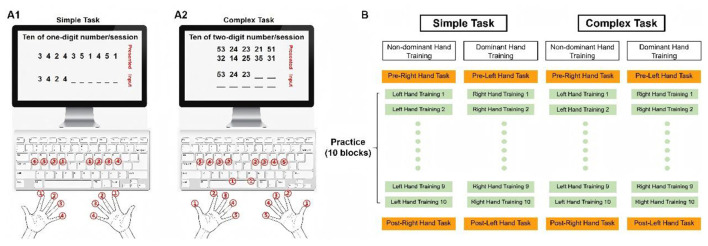
Experimental setup Illustration of task complexity and experimental procedure. **(A1)** In the one-digit (simple) motor task, participants used four fingers (excluding the thumb), with each finger assigned to a single digit. **(A2)** In the two-digit (complex) motor task, all five fingers were utilized, with each digit requiring sequential presses by two designated fingers. **(B)** Participants were randomly assigned to perform either the simple or complex motor task using either their dominant right or non-dominant left hand, yielding four conditions: CL, CR, SL and SR. The training phase comprised 10 blocks. Prior to training, baseline performance of the trained hand was measured using a similar task. Following training, participants repeated this task to assess motor skill acquisition for the trained hand. Colors differentiate the experimental phases: orange indicates pretest and posttest of the trained hand, while gray represents the 10 training blocks. Adapted from Zhao et al. (2025). The experimental setup was reproduced identically for methodological clarity (Zhao et al., 2025).

Consistent with our previous study (Wang et al., 2022), participants performed a one-digit movement task (Figure 1A1) and a two-digit movement task (Figure 1A2) using either their dominant or non-dominant hand. They were instructed to press designated keys with specific fingers corresponding to the numbers displayed on the screen. For the simple one-digit task, participants used four fingers (excluding the thumb), each mapped to a specific digit (index finger: 1, middle finger: 2, ring finger: 3, little finger: 4; Figure 1A1). For the complex two-digit task, all five fingers were used (thumb: 1, index finger: 2, middle finger: 3, ring finger: 4, little finger: 5), requiring sequential key presses with two fingers for each digit pair (Figure 1A2). Each session comprised 10 randomly presented one-digit or two-digit numbers presented without repetition, followed by a 3 sec intermission. Incorrect inputs were highlighted in red and could not be corrected.

A single session was defined as consisting of either 10 one-digit numbers in the simple task or pairs of two unique non-repeating digits in the complex task, with 10 such sessions forming one block. The training phase consisted of 10 blocks, separated by 2 min rest intervals (Figure 1B). Each block had a fixed duration of 2 mins, during which 10 one-digit or two-digit numbers were presented simultaneously in each session, corresponding to the simple and complex tasks, respectively. Participants were instructed to type as quickly and accurately as possible. The program ended when the 2-min limit was reached. Before the training phase, baseline performance was assessed separately using a similar task comprising 10 sessions, equivalent to 100 one-digit or 100 two-digit numbers. After the training phase, a post-training assessment was conducted using the same task as the pretest to evaluate the acquisition of motor skills. The task would stop when 100 one-digit or 100 two-digit numbers were completed. Task completion time (in seconds) and the number of response errors were recorded for each test block. Thus, the experimental protocol consisted of: Pre-test (100 one- or two-digit numbers), followed by 10 training blocks (2 min each), and concluded with a Post-test (100 one- or two-digit numbers). All procedures were completed within a single day. The experimental setup, depicted in Figure 1 adapted from our prior work (Zhao et al., 2025) to ensure consistency.

### EEG recording

EEG signals were recorded using a digital AC amplifier with tin electrodes positioned at 64 scalp sites on an elastic cap (BioSemi, Netherlands). The BioSemi system comprises 64 scalp electrodes and 2 default CMS/DRL channels serving as references, adhering to the 10/20 system. Channels C3 and C4 captured signals from the left and right M1 respectively (Rich and Gillick, 2019; Serra Maia et al., 2023). Impedances for all EEG channels were maintained below ±20 mV. The data were sampled at a rate of 2048 Hz.

### EEG data processing

The EEG data underwent processing using EEGLAB (Delorme and Makeig, 2004), an open-source toolbox within the MATLAB2021b environment. To mitigate hemisphere bias, continuous EEG data were re-referenced to the average of whole-brain signals (Luck, 2005; Nunez et al., 2006). The raw data were subjected to bandpass filtering between 1–80 Hz, and power line noise was eliminated with a notch filter set at 50 Hz. Subsequently, the continuous EEG data were segmented into consecutive, non-overlapping 2 sec epochs. This fixed length epoching was employed to meet the stationarity requirements for spectral analysis, providing a frequency resolution of 0.5 Hz while maintaining sufficient data length for reliable power estimation and subsequent artifact rejection. Independent Component Analysis (ICA) was then applied to remove artifacts arising from muscle activity, eye movements, head movements, heartbeat, and respiration. A Laplace transform was performed using the CSD toolbox (Tenke and Kayser, 2012), which was a spatial filtering technique designed to minimize the influence of common sources and allow us to pinpoint signals with greater accuracy to specific cortical regions.

### Spectral power decomposition

The spectral power decomposition of the EEG signals was calculated over the entire time frame, commencing from the initiation of movement for the trained hands, both before and after training. This analysis was conducted separately for the theta (4-8 Hz), alpha (8-12 Hz), beta (12-30 Hz), low-gamma (30-50 Hz), and high-gamma (50-80 Hz) frequency bands. Based on established literature highlighting the role of gamma oscillations in neuronal excitation, motor unit recruitment, and learning-induced neuroplasticity within the motor cortex (Otsuka and Kawaguchi, 2021; Haverland et al., 2025), our primary analysis focused on the gamma band. Analysis of other frequency bands was conducted for completeness.

### Statistical analyses

Statistical analyses were conducted using the SPSS Statistics Package (version 28.0, SPSS Inc., Chicago, IL). Data normality was assessed using the Shapiro-Wilk test, and the homogeneity of variances was evaluated with Levene's test. Demographic disparities across the four experimental conditions were analyzed using one-way ANOVA, with age and handedness serving as independent variables. For behavioral outcomes, two-way analyses of variance (ANOVA) were first conducted separately to examine differences in completion time and error number at baseline and post-training. The trained hand (left or right) and task complexity (simple or complex) were treated as between-subjects factors, with completion time and error number analyzed as distinct dependent variables. Subsequently, two-way ANOVAs were performed to compare training-related changes in behavioral measures, again with the trained hand and task complexity as between-subjects factors. The changes (post-training minus baseline) for completion time and error number were analyzed as separate dependent variables.

For spectral power analysis, two-way ANOVAs were initially conducted to compare neural activation in bilateral M1 regions in the low-gamma and high-gamma frequency bands at baseline and post-training. The trained hand and task complexity served as between-subjects factors, with low-gamma and high-gamma power analyzed as different dependent variables. Further two-way ANOVAs were employed to examine training-induced changes in iM1 across frequency bands. The trained hand and task complexity were again included as between-subjects factors, while changes (post-training minus baseline) for full-band outcomes (theta, alpha, beta, low-gamma, and high-gamma power) in iM1 were separately analyzed as dependent variables. The assumption of compound symmetry was tested with Mauchly's test, and the Greenhouse-Geisser correction was applied when necessary. Specifically, when the Epsilon value was less than 0.75 for Mauchly's test of sphericity, the Greenhouse-Geisser-corrected value was used; for Epsilon values greater than 0.75, the Huynh-Feldt-corrected value was applied. Additionally, a Bonferroni correction was utilized for multiple comparisons. *Post-hoc* analyses were conducted when significant main or interaction effects were detected. The effect sizes of the independent variables were expressed using eta squared (η^2^). The threshold for statistical significance was set at *p* < 0.05.

Pearson's correlation analyses were performed separately for each condition. First, we assessed the association between baseline behavioral performance and iM1 activation in the high-gamma band during the pre-test session. Subsequently, separate correlation analyses were conducted to examine the relationship between training-induced changes in behavior and changes in iM1 high-gamma activation across the different experimental conditions or pooled together. The significance level was set at *p* < 0.05.

## Results

### Behavioral results

**Figure 2:**
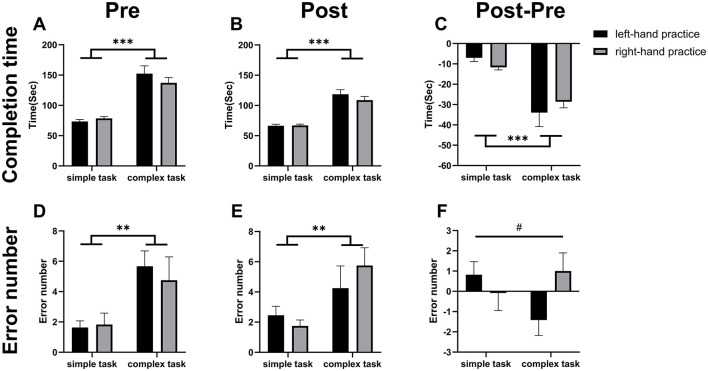
Motor skill acquisition. Results of two-way ANOVA. **(A)** indicates during the pre-test, the completion time was significantly longer in the complex conditions (CL, CR) than in the simple conditions (SL, SR). **(B)** Shows during the post-test, the completion time was significantly longer in the complex conditions than in the simple conditions. **(C)** demonstrates the difference in completion time (post-test minus pre-test) was significantly greater in the complex conditions than in the simple conditions, indicating a significantly larger improvement in the complex conditions. **(D)** reveals during the pre-test, the error number was significantly higher in the complex conditions than in the simple conditions. **(E)** indicates during the post-test, the error number was significantly higher in the complex groups than in the simple conditions. **(F)** shows the difference in error number (post-test minus pre-test) did not differ significantly between the simple and complex conditions. But a significant interaction effect of “Task complexity” × “Hand” was detected (^#^*p* < 0.05). Asterisks denote main effect significant differences: *p < 0.05, **p < 0.01, ***p < 0.001.

We first assessed baseline performance based on the completion time (Figure 2A) and error number (Figure 2D) across four conditions. A significant main effect of task complexity was observed for both the completion time and error number, with the complex task taking longer to complete (144.90 ± 37.65 seconds) compared to the simple task (76.09 ± 10.75 seconds) and more errors in the complex tasks (5.21 ± 4.44 numbers) compared to the simple ones (1.74 ± 2.07 numbers), regardless of the dominance (left vs. right) of the hand (two-way ANOVA, main effect of “Task complexity”, completion time: F_(1, 43)_ = 71.320, *p* < 0.001, η^2^ = 0.624; error number: F _(1, 43)_ = 11,208, *p* = 0.002, η^2^ = 0.207, main effect of “Hand”, completion time: F_(1, 43)_ = 0.377, *p* = 0.543, η^2^ = 0.009; error number: F _(1, 43)_ = 0.120 *p* = 0.730, η^2^ = 0.003, interaction of “Task complexity” x “Hand”, completion time: F _(1, 43)_ = 1.554, *p* = 0.219, η^2^ = 0.035; error number: F _(1, 43)_ = 0.288, *p* = 0.594, η^2^ = 0.007).

Secondly, we quantified completion time end error numbers for each condition after the training (Post) on completion time (Figure 2B) and error number (Figure 2E). A pattern of main effects consistent with that observed at baseline was found only for task complexity, with no significant effects for the main effect of hand or the interaction (two-way ANOVA, main effect of “Task complexity”, completion time: F _(1, 43)_ = 79.848, *p* < 0.001, η^2^ = 0.650; error number: F _(1, 43)_ = 8.032, *p* = 0.007, η^2^ = 0.154, main effect of “Hand”, completion time: F _(1, 43)_ = 0.793, *p* = 0.378, η^2^ = 0.018; error number: F _(1, 43)_ = 0.151 *p* = 0.699, η^2^ = 0.004, interaction of “Task complexity” x “Hand”, completion time: F _(1, 43)_ = 0.956, *p* = 0.334, η^2^ = 0.022; error number: F _(1, 43)_ = 1.162, *p* = 0.287, η^2^ = 0.026).

Finally, between-condition differences in training effect changes were analyzed. For completion time (Figure 2C), a significant main effect of “Task complexity” was observed, but no significant effects were found for “Hand” nor the interaction (two-way ANOVA, main effect of “Task complexity”, F _(1, 43)_ = 30.350, *p* < 0.001, η^2^ = 0.414; main effect of “Hand”, F _(1, 43)_ = 0.007, *p* = 0.935, η^2^ = 0.001; interaction of “Task complexity” x “Hand”, F _(1, 43)_ = 1.598, *p* = 0.213, η^2^ = 0.036). In contrast, for error number (Figure 2F), while no main effects of “Task complexity” or “Hand” were significant, a significant interaction effect was detected (two-way ANOVA, main effect of “Task complexity”, F _(1, 43)_ = 0.511, *p* = 0.479, η^2^ = 0.012; main effect of “Hand”, F _(1, 43)_ = 0.884, *p* = 0.352, η^2^ = 0.020; interaction of “Task complexity” x “Hand”, F _(1, 43)_ = 4.241, *p* = 0.046, η^2^ = 0.090).

### EEG results

**Figure 3:**
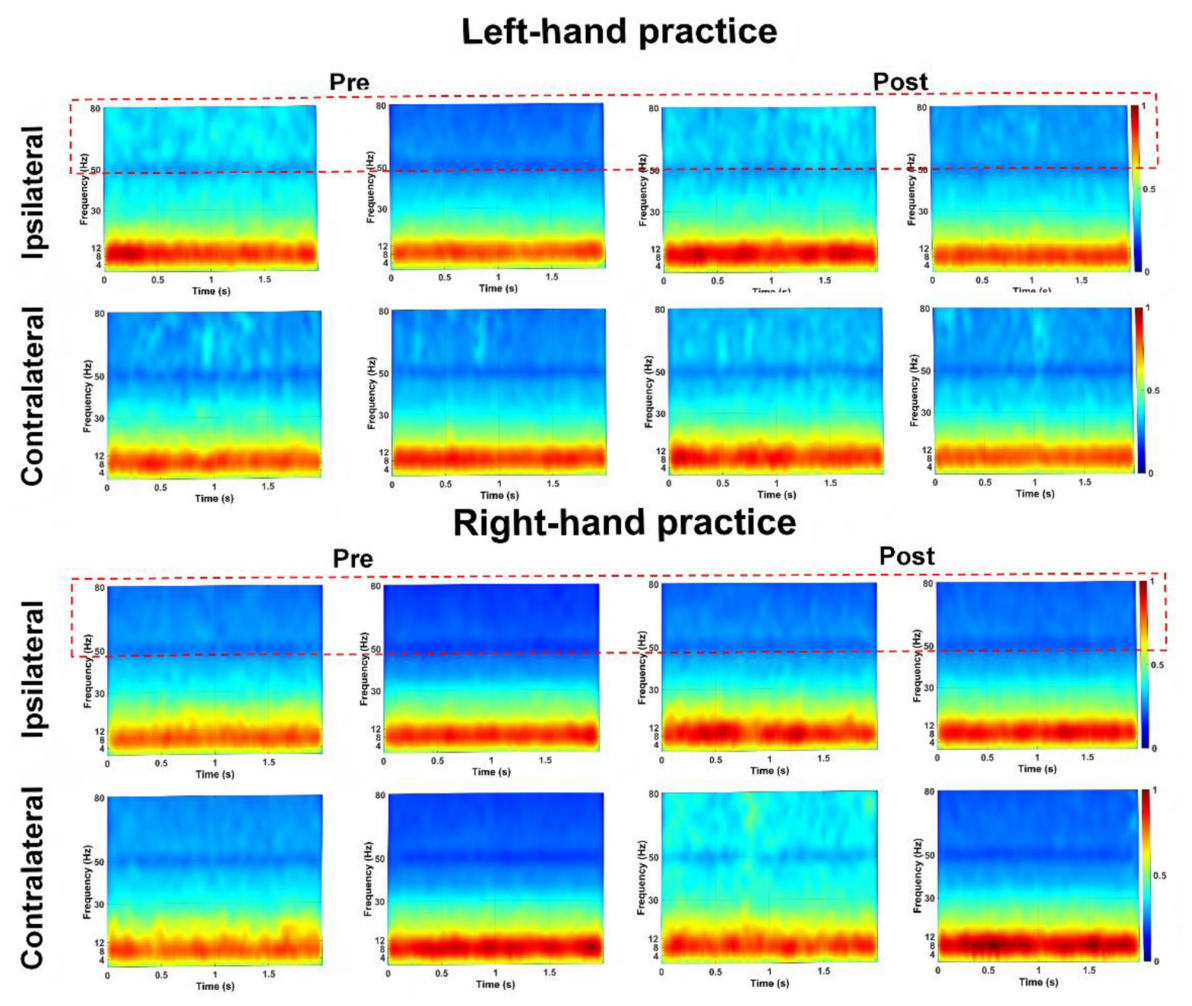
PSD Patterns of each group for ipsilateral vs. contralateral M1 activation during left- and right-hand practice. Illustrating the PSD of ipsilateral and contralateral M1 activation during simple and complex task trials in pre-test and post-test sessions. Results are shown for left-hand practice (top panel) and right-hand practice (bottom panel). The vertical axis represents frequency (0–80 Hz), and the horizontal axis represents time (0–2s). The color bar (range: 0–1) indicates PSD magnitude, with warmer colors (red) denoting higher power values and cooler colors (blue) denoting lower power values. Intermediate values are shown in green. The red dashed box contains the PSD in the range of 50-80 Hz. The image was generated using MATLAB 2021b.

**Figure 4:**
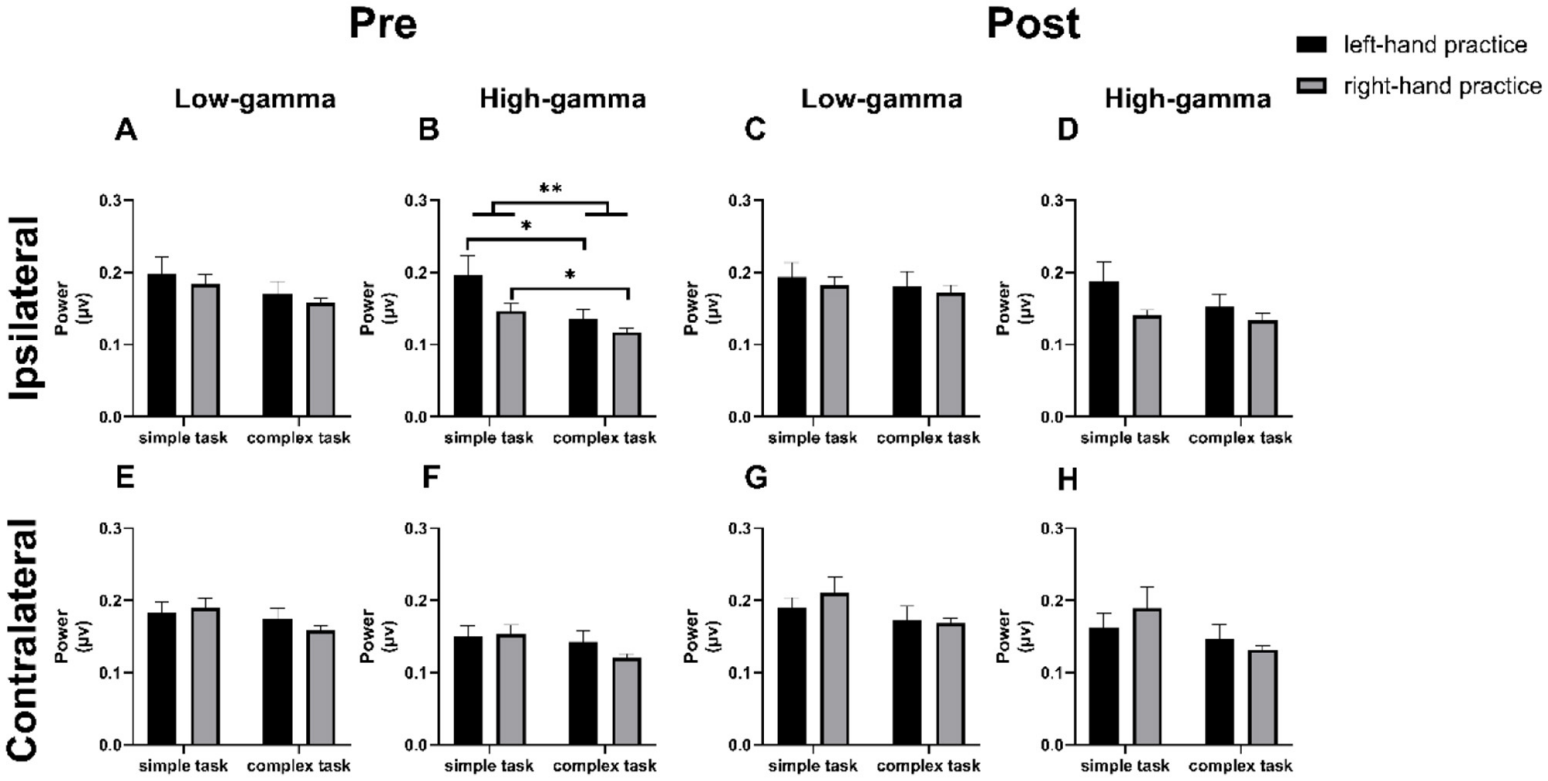
Bilateral M1 Gamma-Band Activation Before and After Practice. **(A)** Pre-test ipsilateral M1 low-gamma activation. **(B)** Pre-test ipsilateral M1 high-gamma activation. **(C)** Post-test ipsilateral M1 low-gamma activation. **(D)** Post-test ipsilateral M1 high-gamma activation. **(E)** Pre-test contralateral M1 low-gamma activation. **(F)** Pre-test contralateral M1 high-gamma activation. **(G)** Post-test contralateral M1 low-gamma activation. **(H)** Post-test contralateral M1 high-gamma activation. Error bars: SEM. ^*^p < 0.05, ^**^p < 0.01.

We conducted a comprehensive analysis of the Power Spectral Density (PSD) recorded from electrodes positioned over the bilateral M1 at pre- and post-tests across four experimental conditions (Figure 3) During pre-tests (for both left-hand and right-hand tests), EEG power in the gamma band over the iM1 was higher during simple tasks compared to complex tasks. However, no significant differences were observed after 10 blocks of training (Figure 4).

Specifically, in the pre-test high-gamma frequency band over the iM1, activation was significantly higher during simple task than during complex tasks (Figure 4B, two-way ANOVA, main effect of “Task complexity”, F _(1, 43)_ = 8.699, *p* = 0.005, η^2^ = 0.152). This effect was consistent between the left-hand and right-hand practice groups (*post-hoc* analysis: SL vs. CL, *p* = 0.047; SR vs. CR, *p* = 0.015). Additionally, a significant main effect of trained hand was observed, indicating differences between the left- and right-hand practice groups (Figure 4B, two-way ANOVA, main effect of “Trained hand”, F _(1, 43)_ = 4.841, *p* = 0.033, η^2^ = 0.085). No significant interaction was found (Figure 4B, two-way ANOVA, interaction of “Task complexity” x “Hand”, F _(1, 43)_ = 1.075, *p* = 0.306, η^2^ = 0.024).

No significant main effects or interaction effects were detected in the low-gamma band (Figure 4A, two-way ANOVA, main effect of “Task complexity”, F _(1, 43)_ = 3.031, *p* = 0.089, η^2^ = 0.066; main effect of “Hand”, F _(1, 43)_ = 0.852, *p* = 0.361, η^2^ = 0.019; interaction of “Task complexity” x “Hand”, F _(1, 43)_ = 0.036, *p* = 0.970, η^2^ = 0.007), over the cM1 (Figures 4E, F, two-way ANOVA, main effect of “Task complexity”, low-gamma: F _(1, 43)_ = 2.320, *p* = 0.135, η^2^ = 0.051; high-gamma: F _(1, 43)_ = 2.514, *p* = 0.120, η^2^ = 0.055, main effect of “Hand”, low-gamma: F _(1, 43)_ = 0.105, *p* = 0.747, η^2^ = 0.002; high-gamma: F _(1, 43)_ = 0.480 *p* = 0.492, η^2^ = 0.011, interaction of “Task complexity” x “Hand”, low-gamma: F _(1, 43)_ = 0.723, *p* = 0.400, η^2^ = 0.017; high-gamma: F _(1, 43)_ = 1.042, *p* = 0.313, η^2^ = 0.024), or in any frequency band of the ipsilateral or contralateral M1 during post-training (Figures 4C, D, G, H, two-way ANOVA, main effect of “Task complexity”, iM1 in low-gamma: F _(1, 43)_ = 0.502, *p* = 0.482, η^2^ = 0.012; iM1 in high-gamma: F _(1, 43)_ = 1.512, *p* = 0.226, η^2^ = 0.034; cM1 in low-gamma: F _(1, 43)_ = 3.175, *p* = 0.082, η^2^ = 0.069; cM1 in high-gamma: F _(1, 43)_ = 3.152, *p* = 0.083, η^2^ = 0.068, main effect of “Hand”, iM1 in low-gamma: F _(1, 43)_ = 0.414, *p* = 0.523, η^2^ = 0.010; iM1 in high-gamma: F _(1, 43)_ = 3.537, *p* = 0.067, η^2^ = 0.076; cM1 in low-gamma: F _(1, 43)_ = 0.252, *p* = 0.618, η^2^ = 0.006; cM1 in high-gamma: F _(1, 43)_ = 0.071, *p* = 0.792, η^2^ = 0.002, interaction of “Task complexity” x “Hand”, iM1 in low-gamma: F _(1, 43)_ = 0.004, *p* = 0.949, η^2^ = 0.002; iM1 in high-gamma: F _(1, 43)_ = 0.641, *p* = 0.428, η^2^ = 0.015; cM1 in low-gamma: F _(1, 43)_ = 0.610, *p* = 0.439, η^2^ = 0.014; cM1 in high-gamma: F _(1, 43)_ = 1.066, *p* = 0.308, η^2^ = 0.024).

**Figure 5:**
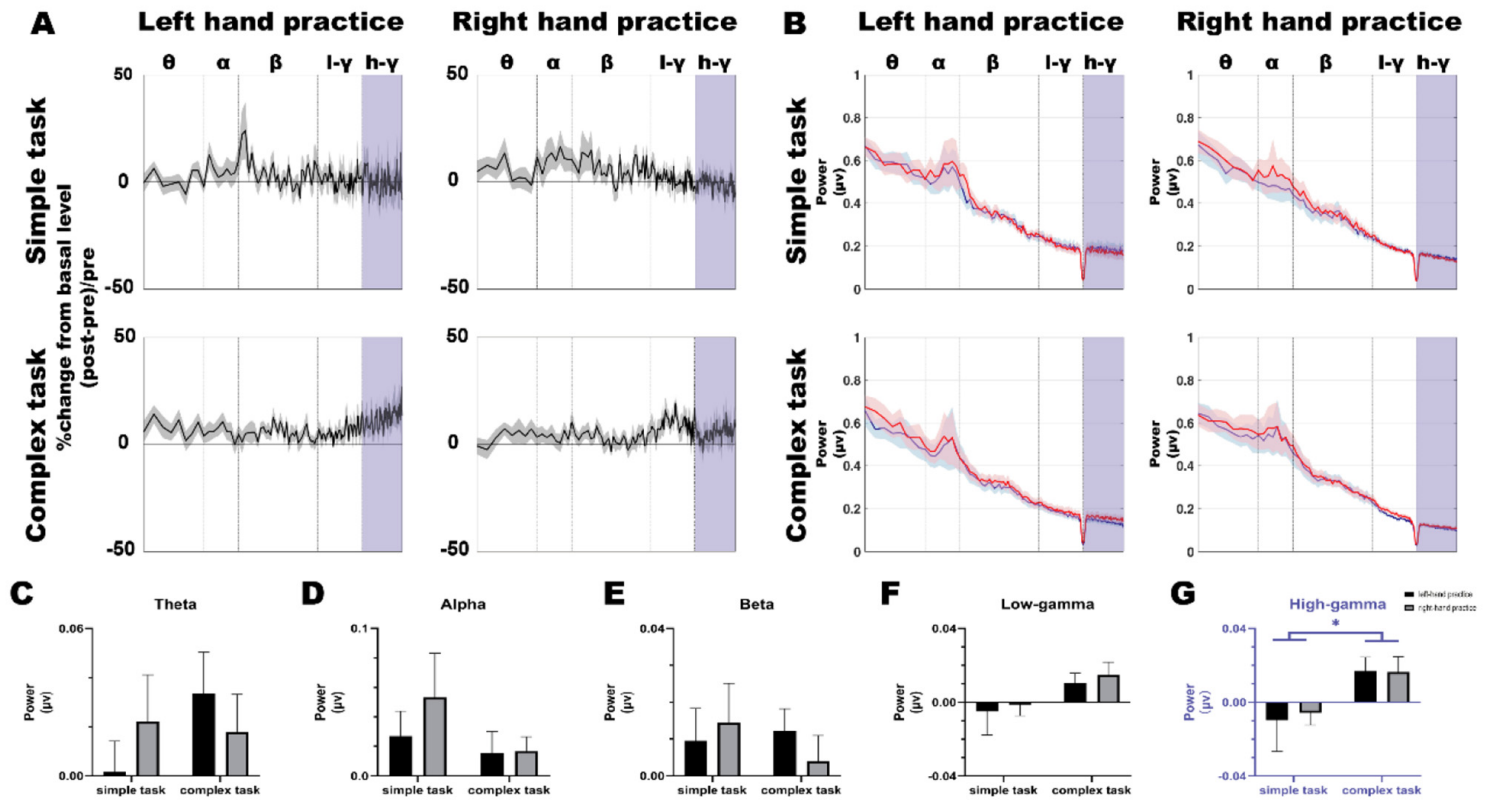
EEG band power changes in iM1 following various practice protocols. **(A)** depicts the percentage distribution changes of iM1 activation intensity after training relative to that before training across different frequency bands, for the four conditions (SL, SR, CL, CR). The percentage were calculated as [(*post-test* value – *pre-test* value)/pre-test value]. The purple areas are used to highlight the 50–80 Hz frequency band area. **(B)** shows the distribution of iM1 power values across different frequency bands before training (blue line) and after training (red line) under the four conditions; the shaded areas represent the SEM. **(C–G)** represent the statistical results of two-way ANOVA for the differences in iM1 activation intensity (post-training value – pre-training value) across different frequency bands in the four conditions. The power value greater than 0 indicates that iM1 activation intensity after training is greater than before training, whereas the power value less than 0 indicates that iM1 activation intensity after training is lower than that before training. **(G)** depicts that in the 50–80 Hz frequency band, the iM1 activation changes (*post-test* value – *pre-test* value) in the complex conditions (CL, CR) show significant differences compared with those in the simple conditions (SL, SR) (^*^p < 0.05). Asterisks indicate statistically significant differences: ^*^p < 0.05.

Finally, analysis revealed distinct patterns in iM1 activation between pre- and post-test (Figures 5A, B). Notably, the complex condition showed a significant increase in high-gamma band activity (Figure 5G, two-way ANOVA, main effect of “Task complexity”, F _(1, 43)_ = 5.530, *p* = 0.023, η^2^ = 0.114; main effect of “Hand”, F _(1, 43)_ = 0.025, *p* = 0.875, η^2^ = 0.001; interaction of “Task complexity” x “Hand”, F _(1, 43)_ = 0.047, *p* = 0.830, η^2^ = 0.001). No significant changes were observed in other frequency bands (Figures 5C–F, two-way ANOVA, main effect of “Task complexity”, theta: F _(1, 43)_ = 0.712, *p* = 0.403, η^2^ = 0.016; alpha: F _(1, 43)_ = 1.512, *p* = 0.226, η^2^ = 0.034; beta: F _(1, 43)_ = 0.224, *p* = 0.638, η^2^ = 0.005; low-gamma: F _(1, 43)_ = 3.890, *p* = 0.055, η^2^ = 0.083, main effect of “Hand”, theta: F _(1, 43)_ = 0.024, *p* = 0.878, η^2^ = 0.001; alpha: F _(1, 43)_ = 0.502, *p* = 0.482, η^2^ = 0.012; beta: F _(1, 43)_ = 0.036, *p* = 0.851, η^2^ = 0.001; low-gamma: F _(1, 43)_ = 0.268, *p* = 0.607, η^2^ = 0.006, interaction of “Task complexity” x “Hand”, theta: F _(1, 43)_ = 1.259, *p* = 0.268, η^2^ = 0.028; alpha: F _(1, 43)_ = 0.407, *p* = 0.527, η^2^ = 0.009; beta: F _(1, 43)_ = 0.633, *p* = 0.431, η^2^ = 0.015; low-gamma: F _(1, 43)_ = 0.003, *p* = 0.958, η^2^ = 0.001).

### Correlation results

To investigate the relationship between neural activity and behavior, we performed a series of Pearson correlation analyses. The simple and complex task groups were analyzed separately initially, as they were pooled for this analysis due to the absence of a main effect of hand. First, we examined the association at baseline between behavioral performance and iM1 high-gamma activation. No significant correlation was found between baseline completion time and baseline iM1 high-gamma power in any condition (Supplementary Figures 1A, B, Pearson's Correlation test, simple condition: *r* = 0.228, *p* = 0.296, CI95 = [-0.20, 0.59]; complex condition: *r* = 0.341, *p* = 0.103, CI95 = [-0.07, 0.65]). Subsequently, we analyzed the relationship between the training-induced changes (*post-pre*) in high-gamma power in the iM1 and the changes in completion time. No significant correlation was found in either the simple or the complex conditions (Supplementary Figures 1C, D, Pearson's Correlation test, simple condition: *r* = 0.071, *p* = 0.748, CI95 = [-0.35, 0.47]; complex condition: *r* = −0.273, *p* = 0.196, CI95 = [-0.61, 0.15]). However, a subsequent analysis that combined all participants from all four groups, irrespective of training hand or task complexity, revealed a significant negative correlation (Supplementary Figure 1E, Pearson's Correlation test, r = −0.305, *p* = 0.037, CI95 = [-0.54, -0.02]).

## Discussion

This study aimed to address a critical gap in motor neuroscience: how the electrodes over cM1 and iM1 activation dynamics (specifically in gamma frequency bands) evolve during the acquisition of simple vs. complex motor skills, and whether these dynamics are modulated by hand dominance. Our hypothesis predicted that the electrodes over cM1 activation would remain stable across training, while iM1 activation would show task-dependent plasticity (enhanced post-training for complex but not simple tasks). The findings partially validate this framework, confirming task-dependent iM1 plasticity in high-gamma bands, while revealing unexpected gamma sub-band specificity and hand dominance effects that extend current understanding of motor skill acquisition mechanisms.

First, our core results align with the hypothesis: following 10 training sessions, iM1 activation in the high-gamma band increased for complex tasks but decreased for the simple task with both hands, eliminating the pre-training difference between simple and complex tasks, while cM1 activation remained unchanged. These findings provide support for the mechanism proposed in our hypothesis, that simple tasks readily develop automatic processing through repetition (Schneider and Chein, 2003). This also supports the idea that iM1 plays a dynamic, task-dependent role in skill acquisition, consistent with prior proposals that iM1 activation scales with task demands (Winstein et al., 1997; Hummel et al., 2003; Verstynen et al., 2005; Holper et al., 2009; Buetefisch et al., 2014). But extends this by demonstrating that such plasticity is specific to task-induced changes rather than static task properties. Notably, the stability of cM1 activation aligns with its established role as a core executor of motor commands (Tanji et al., 1988; Catalan et al., 1998; Bütefisch et al., 2005), reinforcing that its function is less dependent on the training stage for well-practiced movements.

Crucially, our results may explain the long-standing contradictions in the literature regarding iM1's role in learning. Dafotakis et al. (2008) reported suppressed iM1 excitability alongside improved performance, while van den Berg et al. (2011) found iM1 disruption impairs complex ipsilateral tasks. Our dynamic tracking (pre- vs. post-training) clarifies this tension: iM1 activation is not universally facilitatory or inhibitory but depends on task complexity. Simple tasks, which may rely primarily on cM1-mediated execution, show iM1 suppression after training, which is consistent with Dafotakis et al. (2008) observation of suppressed iM1 in simple learning contexts. In contrast, complex tasks demand interhemispheric coordination, driving iM1 high-gamma enhancement post-training, supporting van den Berg et al. (2011) proposal that iM1 contributes to complex motor control. This task-dependent, dynamic pattern may explain why prior studies, relying on static measurements or conflating task types, yielded conflicting results.

In line with previous studies, scalp EEG records cortical oscillations from neural sources. These oscillations span a range of frequencies and are conventionally divided into two subcategories of gamma: low gamma (30-50 Hz) and high gamma (50-80 Hz) (Li et al., 2015; Shinozaki et al., 2016). Low gamma generally occurs in basic cognitive processes, such as the 30-50 Hz oscillations observed in working memory tasks (Barr et al., 2011). In contrast, high gamma typically refers to oscillations above 50 Hz, which are only present in high-level cognitive tasks that require fine-grained information processing. Examples include in visual delayed discrimination tasks (Haenschel et al., 2009). This also provides a good explanation for our findings: stronger high-gamma band activation during complex tasks, which may require greater cognitive engagement. Notably, there is no unified definition of the frequency range of gamma oscillations. In some intracranial EEG studies, frequencies of up to 250 Hz can be captured (Manssuer et al., 2024); however, scalp EEG studies usually struggle to capture such high-frequency ranges.

The functional role of this ipsilateral response may involve compensatory support that, when tasks are highly complex or the contralateral hemisphere is untrained, the ipsilateral motor cortex might help shape appropriate motor patterns via excitatory and inhibitory connections (Verstynen et al., 2005; Buetefisch et al., 2011). Consequently, performance differences between hands correlated with hemispheric asymmetry in ipsilateral activation, with the strongest activation occurring during use of the less proficient hand to balance interhemispheric communication, and this is supported by prior findings of increased ipsilateral activation with task complexity (Verstynen et al., 2005; Holper et al., 2009). Paradoxically, however, our data indicate that simple sequence tasks within the gamma band elicited stronger ipsilateral activation compared to complex sequences during the pre-test. We propose two non-mutually exclusive explanations: (1) Movement intensity, as simple tasks were executed faster with higher keypress frequency, consistent with previous (Pfurtscheller et al., 2003) findings that gamma power correlates with movement rate. (2) Interhemispheric inhibition: untrained complex tasks may trigger stronger cM1-to-iM1 inhibition (Kobayashi et al., 2009), reducing net cM1 activation measurable via EEG. Post-training, this inhibition diminishes, allowing iM1 activation to rise without requiring increased cM1 output, a pattern mirrored in Suzuki et al.'s (2013) observation of reduced iM1 excitability in untrained states. This is also consistent with the results we observed.

Our correlational analyses reveal a complex relationship between motor learning and iM1 neuroplasticity. The absence of significant correlations within individual conditions is likely due to limited statistical power and inter-individual variability across subgroups. However, pooling all groups revealed a significant negative correlation, indicating that greater increases in iM1 high-gamma activity were associated with greater behavioral improvement across participants, regardless of task complexity or trained hand. This suggests that iM1 high-gamma activity may reflect a general neuro plastic mechanism supporting motor learning, though its expression varies within specific conditions.

In summary, this study clarifies that iM1's role in motor skill acquisition may be neither universally facilitatory nor inhibitory but also depends on task complexity, hand dominance, and gamma sub-band. By resolving prior contradictions and linking gamma dynamics to plasticity, we advance a more precise framework for understanding how the motor system adapts to skill acquisition.

## Limitation

Limitations include the focus on gamma bands, leaving alpha/beta dynamics underexplored, and the use of right-handed participants, which limits generalizability to left-handed populations. Additionally, EEG's spatial resolution prevents precise localization of gamma activity within M1 subregions. Future work could combine fMRI-EEG fusion to enhance spatial precision and use gamma-tACS to test causal relationships, for example, whether boosting 50–80 Hz iM1 activity accelerates non-dominant hand complex learning. Lastly, only finger tasks were used, and the potential actions of hands are complex, it is a representation of broader action possibilities.

## Conclusion

In the present study, we examined the underlying neural mechanisms of motor skill acquisition considering task complexity. Our data indicates that task complexity affects iM1 activation. Specifically, CL and CR increased the high-gamma frequency band. These findings highlight differential neural responses within specific frequency bands, potentially reflecting distinct impacts of the interventions applied to each group.

## Data Availability

The raw data supporting the conclusions of this article will be made available by the authors, without undue reservation.
