# Supplementary material for: EEG activity over ipsilateral and contralateral M1 during simple and complex hand tasks: variations with motor learning
Source: Front Neurosci. 2025 Nov 6;19:1681250. doi: 10.3389/fnins.2025.1681250 (PMC12631381; doi:10.3389/fnins.2025.1681250)
Supplement: Supplementary file 1 [file Supplementary_file_1.docx]

**Supplementary Materials**


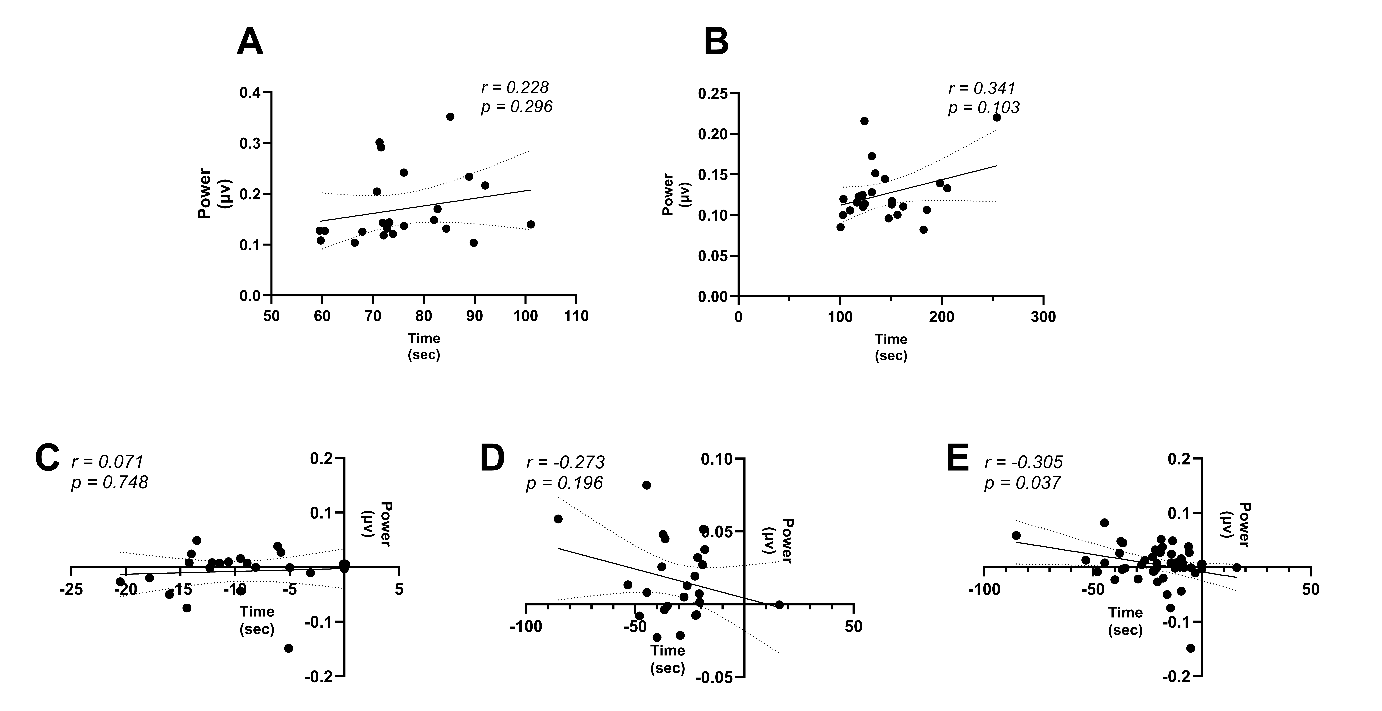


**Supplementary Fig. 1. Correlations between neural activity and behavior. A** and **B** Scatter plots show the correlation between baseline completion time and baseline iM1 high-gamma power for the simple (Fig. A) and complex (Fig. B) conditions. **C** and **D** Scatter plots of the relationship between training-induced changes (post-pre) in completion time and iM1 high-gamma power for the simple (Fig. C) and complex (Fig. D) conditions. **E** represents a significant negative correlation between neural and behavioral changes when all groups were pooled together. Solid lines represent the regression fit; and dashed lines indicate the 95% confidence interval. Each data point represents an individual participant.
